# Deletion of *HIF-1α* partially rescues the abnormal hyaloid vascular system in *Cited2* conditional knockout mouse eyes

**Published:** 2012-05-11

**Authors:** Tai-Qin Huang, Yiwei Wang, Quteba Ebrahem, Yu Chen, Cindy Cheng, Yong Qiu Doughman, Michiko Watanabe, Sally L. Dunwoodie, Yu-Chung Yang

**Affiliations:** 1 Department of Biochemistry and Cancer Center, Case Western Reserve University School of Medicine, Cleveland, OH; 2 Department of Ophthalmology, Cole Eye Institute, Cleveland Clinic Lerner College of Medicine, Cleveland, OH; 3 Department of Pharmacology, Rainbow Babies' and Children's Hospital, Case Western Reserve University School of Medicine, Cleveland, OH; 4 Department of Pediatrics, Rainbow Babies' and Children's Hospital, Case Western Reserve University School of Medicine, Cleveland, OH; 5 Developmental and Stem Cell Biology Division, The Victor Chang Cardiac Research Institute, Darlinghurst, NSW. St Vincent’s Clinical School University of New South Wales, Kensington, NSW, Australia

## Abstract

**Purpose:**

Cited2 (CBP/p300-interacting transactivators with glutamic acid (E) and aspartic acid (D)-rich tail 2) is a member of a new family of transcriptional modulators. *Cited2* null embryos exhibit hyaloid hypercellularity consisting of aberrant vasculature in the eye. The purpose of the study is to address whether abnormal lenticular development is a primary defect of *Cited2* deletion and whether deletion of hypoxia inducible factor (HIF)-1α or an HIF-1α target gene, vascular endothelial growth factor (*VEGF*), could rescue abnormal hyaloid vascular system (HVS) in *Cited2* deficient adult eyes.

**Methods:**

Le-Cre specific *Cited2* knockout (*Cited2*^CKO^) mice with or without deletion of *HIF-1α* or *VEGF* were generated by standard Cre-Lox methods. Eyes collected from six-eight weeks old mice were characterized by Real Time PCR and immunohistological staining.

**Results:**

*Cited2*^CKO^ mice had smaller lenses, abnormal lens stalk formation, and failed regression of the HVS in the adult eye. The eye phenotype had features similar to persistent hyperplastic primary vitreous (PHPV), a human congenital eye disorder leading to abnormal lenticular development. Deletion of *HIF-1α* or *VEGF* in *Cited2* knockout eyes partially rescued the abnormal HVS but had no effect on the smaller lens and abnormal lens stalk differentiation. Intravitreal injection of Topotecan (TPT), a compound that inhibits HIF-1α expression, partially eliminated HVS defects in *Cited2*^CKO^ lenses.

**Conclusions:**

Abnormal HVS is a primary defect in *Cited2* knockout mice, resulting in part from dysregulated functions of HIF-1 and *VEGF*. The *Cited2*^CKO^ mouse line could be used as a novel disease model for PHPV and as an in vivo model for testing potential HIF-1 inhibitors.

## Introduction

The lens consists of the lens capsule, the lens epithelium, and the lens fibers. The lens capsule is a collagen containing basement membrane structure that completely surrounds the lens. The lens epithelium is a simple cuboidal epithelium between the lens capsule and the lens fibers in the anterior portion of the lens. The lens fibers are long, thin, transparent cells filled with crystalline proteins to ensure lens transparency [1]. Lens development begins in the ectoderm with formation of the lens placode. In the vertebrate eye, lens development is accompanied by the growth of the surrounding capillary network. This network is found within the anterior papillary membrane (APM) on the anterior surface of the lens and includes the tunica vasculosalentis (TVL) posterior to the lens and provides nutrients to intraocular components. The fetal vasculature normally regresses starting late in fetal life and completes regression in the first two weeks after birth in rodents [2]. After vascular regression, the lens becomes transparent and resides in a hypoxic environment. However, the role of hypoxia involved in lens development remains elusive.

One of the major transcription factors governing hypoxic responses is HIF-1, which is a heterodimeric protein composed of HIF-1α and HIF-1β subunits [3]. HIF-1α is degraded through a proteasome-mediated pathway under normoxia but stabilized under hypoxia [4]. Stabilized HIF-1α dimerizes with HIF-1β, binds to gene promoters containing hypoxia-response elements (HREs) and recruits transcriptional coactivators such as CREB-binding protein (CBP), E1A binding protein p300 (p300), or steroid receptor coactivator-1 (SRC-1) [5-7]. Although hypoxia has been implicated in controlling normal homeostasis and pathological conditions, the molecular mechanism of how HIF-1α interacts with transcription cofactors to initiate target gene expression is not clearly understood. As a coactivator, CBP/p300 interacts with HIF-1α through its cysteine/histidine-rich 1 (CH1) domain to modulate HIF-1 target gene expression [6].

Recently, we and others found that Cited2 (CBP/p300-interacting transactivator, with glutamic acid (E) and aspartic acid (D)-rich tail 2), a transcriptional modulator, is a negative regulator for HIF-1 through its competitive binding with HIF-1α to the CH1 domain of CBP/p300 [8,9]. It was first demonstrated by Bhattacharya et al. [10] based on in vitro transfection studies that a p300 CH1 mutant peptide, defective in HIF-1α but not Cited2 binding, enhanced endogenous HIF-1 function. This notion was further supported by the similar nuclear magnetic resonance (NMR) structures of HIF-1α/p300-CH1 and Cited2/p300-CH1 complexes and the identification of the LPXL motif in both HIF-1α and Cited2 as the basis for the competition [8]. In our studies, we showed that Cited2 is a negative regulator of HIF-1 in embryonic heart [9,11] and eye [12] using *Cited2* knockout mice. The transcript levels of HIF-1 target genes such as phosphoglycerate kinase 1 (*PGK1*), glucose transporter 1 (*Glut1*), and vascular endothelial growth factor (*VEGF*) were highly expressed in the *Cited2*^−/−^embryonic heart [9]. Furthermore, embryonic heart defects could be rescued by *HIF-1α* heterozygosity in *Cited2*^−/−^embryos [11]. Similarly, deletion of *HIF-1α* in the lens specifically eliminated the hypercellularity and aberrant structure of the hyaloid vasculature in *Cited2*^−/−^ embryonic eyes [12]. We therefore demonstrated that Cited2 exerts a unique feedback regulatory mechanism to limit excess HIF-1 activation and to maintain normal tissue homeostasis.

*VEGF* is one of the HIF-1target genes involved in early vascular development and angiogenesis. It functions by binding to the transmembrane tyrosine kinase receptor vascular endothelial growth factor receptor-1 (VEGFR1; Flt-1) and VEGFR2 (Flk-1, KDR) on the cell surface. Deletion of one allele of *VEGF* or disruption of *VEGFR2* leads to embryonic lethality [13]. *VEGF* is expressed in mouse lens epithelial and fiber cells. Overexpression of *VEGF* in the mouse lens induces microphthalmia, hypertrophy, and persistence of the hyaloid vasculature [14]. Lenses lacking VEGF are smaller in size with mild nuclear opacities that regress with age [15]. Transgenic mice overexpressing stable forms of HIF-1α in lens epithelial cells have smaller lenses at birth and the tunica vasculosa lentis (TVL) do not form, although the biologic consequences of HIF-1 overexpression or hyperactivation on the hyaloid vasculature have not been demonstrated [16].

Persistent hyperplastic primary vitreous (PHPV), also known as persistent fetal vasculature, is a congenital abnormality of the eye caused by the failure of regression of the primary vitreous [17]. During embryogenesis of human eye, nutrients are provided by a hyaloid artery between the retina and crystalline lens, which is later replaced by the developing retinal vasculature.

However, failure of regression of the primary vitreous during third to ninth months of gestation causes PHPV [17,18]. In most case, PHPV is sporadic and unilateral while bilateral PHPV is rare [19]. The disease is complicated and often associated with other ocular abnormalities. The conditions that may mimic PHPV include microphthalmia, congenital cataract, corneal opacity, uveal coloboma, and retinal degeneration [17]. Although several genes, such as protein 53 (*p53*) [20], alternative reading frame (*Arf*) [21,22], norrie disease pseudoglioma (*NDP*) [23], and genes in the int and wg (wingless; *Wnt*) [24] signaling pathway have been implicated in the development of PHPV, the exact mechanisms have not been resolved.

We previously reported lens stalk formation and hyaloid hypercellularity in *Cited2* knockout mouse embryos, most likely through dysregulated HIF-1 function. To address whether the phenotype is a primary defect in the lens, we generated tissue-specific *Cited2* knockout mice. Since deletion of *HIF-1α* partially rescues hyaloid hypercellularity and aberrant vasculature in *Cited2* knockout embryos, we also tested the role of HIF-1 and its target gene *VEGF* in lens development by generating compound mice in which *Cited2* and *HIF-1α* or *VEGF* were deleted in the lens. Based on the fact that Cited2 is a negative regulator of HIF-1, we explored the possibility that the *Cited2*^CKO^ mouse could be an in vivo model for testing potential HIF-1 inhibitors that may be useful for therapies in several clinical settings.

## Methods

### Animals

*Cited2*^flox/flox^ (*Cited2*^fl/fl^) mice (*Cited2*^tm1.1Dunw^ [25]); with C57BL6 genetic background were mated with Le-Cre^+/−^mice [26] to generate *Cited2*^fl/fl^;Le-Cre^-^ and *Cited2*^fl/fl^;Le-Cre^+^ (referred to as *Cited2*^CKO^) mice. To generate Le-Cre specific *Cited2* and *HIF-1α* knockout mice, *Cited2*^fl/fl^;*HIF-1α*^fl/+^ mice were mated with *Cited2*^fl/fl^;*HIF-1α*^fl/fl^;Le-Cre^+^ mice. Mice with deletion of *Cited2* and *VEGF* in the lens were generated by mating *Cited2*^fl/fl^;*VEGF*^fl/+^ mice with *Cited2*^fl/fl^;*VEGF*^fl/+^;Le-Cre^+/−^. Primers for genotyping were: *Cited2*-flox: antisense (a), 5′-CTG CTG CTG CTG GTG ATG AT-3′ and sense(s), 5′-GTC TCA GCG TCT GCT CGT TT-3′; Le-Cre: (a), 5′-GCA TTA CCG GTC GAT GCA ACG AGT GAT GAG-3′ and (s), 5′-GAG TGA ACG AAC CTG GTC GAA ATC AGT GCG-3′; *HIF-1α*-flox: (a), 5′-ATA TGC TCT TAT GAA GGG GCC TAT GGA GGC-3′ and (s), 5′-GAT CTT TCC GAG GAC CTG GAT TCA ATT CCC-3′; *VEGF*-flox: (a), 5′-ACA CAG GAC GGC TTG AAG AT-3′ and (S), 5′-CTA CTG CCG TCC GAT TGA GA-3′.

### Histology

Eyes were removed from euthanized mice and fixed in 10% formalin at 4 ^0^C for 24 h. Transverse 7 μm paraffin embedded sections were collected, hematoxylin-eosin (H&E) stained and examined by light microscopy.

### Immunofluorescence staining

Eyes were removed and mounted in optimal cutting temperature (OCT) immediately following sacrifice of the animal. Five μm frozen sections were fixed in 4% paraformaldehyde/PBS for 10 min and blocked with 10% normal goat serum (NGS) and 0.1% Triton X-100 in PBS for 30 min at room temperature. Sections were incubated with primary antibodies in a humidified chamber overnight at 4 ^0^C. Immunostaining was performed by applying antibodies against Cited2 (Santa Cruz, Santa Cruz, CA or R& D, Minneapolis, MN), α-smooth muscle actin (α-SMA; Sigma, St. Louis, MO), F4/80 (AbDSerotec, Raleigh, NC), and CD31 (BD PharMingen, San Jose, CA) and detected using Cy3-conjugated anti-mouse or anti-rat secondary antibody. Digital images were collected using a Leica DMLB fluorescence microscope (Buffalo Grove, IL).

### Pimonidazole hydrochloride (PIM) staining

Six-week old mice were treated with PIM (20 mg/kg bodyweight; Hypoxyprobe^TM^-1; Chemicon, Billerica, MA) by intravenous (i.v.) injection. Two hours after injection, mice were sacrificed and the eyes were immediately embedded in OCT. Five μm thick sections were used for immunostaining with FITC-conjugated Mab1 antibody (Hypoxyprobe, Inc. Burlington, MA).

### Real Time PCR

Total RNA was extracted from lens using the RNeasy Kit (Qiagen, Valencia, CA) and reverse transcribed into cDNA using SuperScript First-Strand Synthesis System for RT–PCR Kit (Invitrogen, Grand Island, NY). Real-Time PCR was performed by using primers for *Cited2*: sense (s) 5′-CGC ATC ATC ACC AGC AGC AG-3′ and antisense (a) 5′-CGC TCG TGG CAT TCA TGT TG-3′; *HIF-1α*: (s) 5′-GGA CGA TGA ACA TCA AGT CAG-3′ and (a) 5′-GGA ATG GGT TCA CAA ATC AGC-3′; *VEGF*: (s) 5′-ATC TTC AAG CCG TCC TGT GT-3′ and (a) 5′-CTG CAT GGT GAT GTT GCT CT-3′; 18S: (s) 5′-CGT CTG CCC TAT CAA CTT TCG-3′ and (a)5′-CCT TGG ATG TGG TAG CCG TT-3′. Real-Time PCR was performed using iQTM SYBR Green Supermix PCR kit and iCycler machine (Bio-Rad, Hercules, CA). Cycling conditions were 95 °C for 10 min, and 40 cycles of 95 °C for 15 s and 60 °C for 30 s. A melting curve analysis of products was performed routinely following the amplification to test the specificity of the PCR products. 18S was used as an internal control for normalization. After normalization with 18S, average was calculated for each group and control in each group was set as one.

### Intravitreal injection

Six- to eight-week old *Cited2*^fl/fl^;Le-Cre^-^ and *Cited2*^CKO^ mice received intravitreal injection of Topotecan (TPT; Sigma) using a 33-gauge needle. TPT was dissolved in dimethyl sulfoxide (DMSO) and diluted with saline to 20 μg/μl and 40 μg/μl and 2.5 μl was injected into each eye. In each mouse, the left eye was treated with TPT in saline; the right eye was treated with saline containing the same portion of DMSO to minimize the effect of DMSO used to dissolve TPT. Right eye was used as a negative vehicle control. After injection, mouse eyes were covered with Gentak ointment (Akorn, Inc. Lake Forest, IL) to prevent inflammation. After 3 weeks, injected mice were euthanized and eyes were collected for histological analysis.

## Results

### *Cited2* is important in HVS formation and regression in the lens

**Figure 1:**
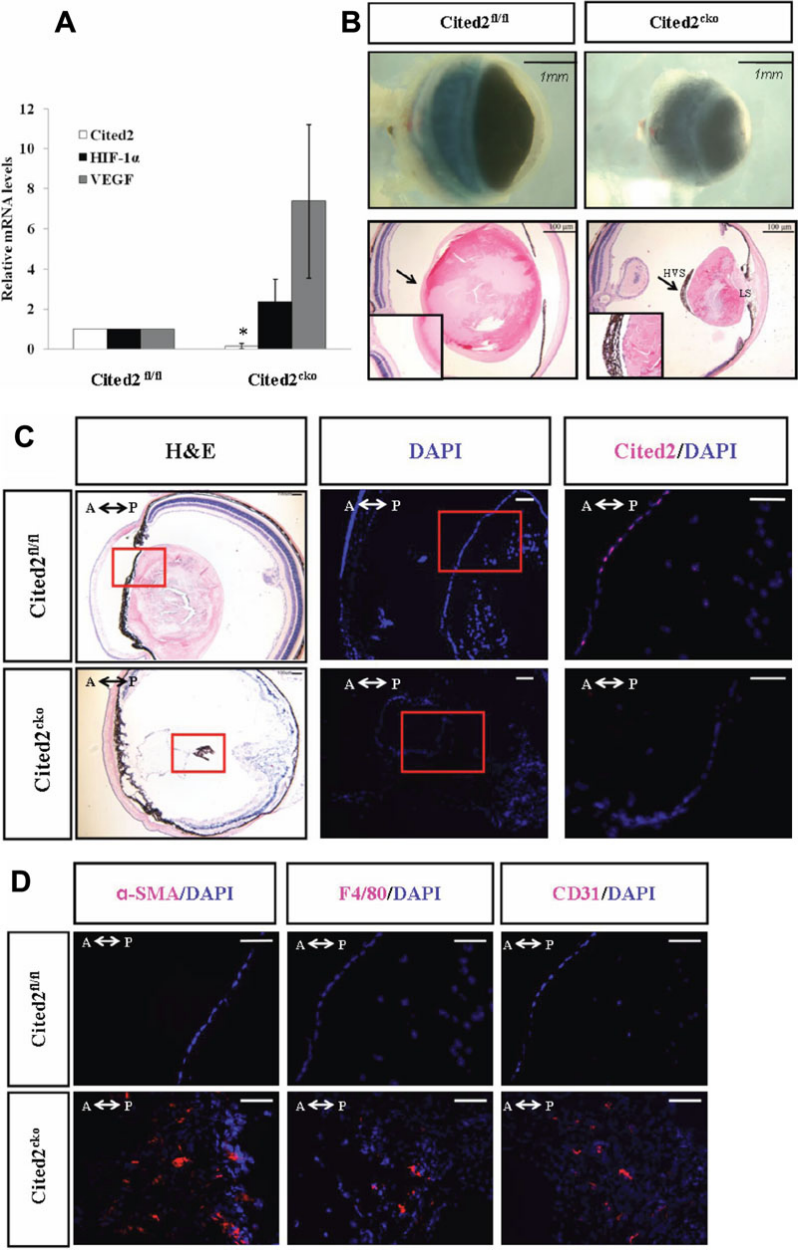
Le-Cre mediated deletion of *Cited2* and morphological changes in *Cited2*^CKO^ mice. **A**: Expression of *Cited2* was decreased in *Cited2*^CKO^ mice compared to control (n=3, *p<0.05). Modest increase of *HIF-1α* and substantial increase of *VEGF* were observed in *Cited2*^CKO^ mice. **B**: Under a dissecting microscope, eyes collected from *Cited2*^CKO^ mice showed smaller sizes and cornea opacity (upper panel). H&E staining showed smaller lens, lens stalk (LS) formation at the anterior side of the lens, and hyaloid hypercellularity and aberrant vasculature at the posterior side of the lens in *Cited2*^CKO^ mice (lower panel). The insets represent higher magnification of the areas indicated by arrows. **C**: Immunostaining with Cited2 antibody showed decreased expression of Cited2 (magenta) in *Cited2*^CKO^ mouse lens. Counterstaining with DAPI (blue) indicated that Cited2 localized in the nucleus. H&E pictures were taken at 5× magnification to show the entire eye structure. DAPI pictures were taken at 10× using adjacent sections. Red box indicates the area shown in the Cited2/DAPI counterstained pictures (20×). Scale bar in each picture indicates 100 μm. **D**: Immunostaining with α-SMA, F4/80, and CD31 (red) showed composition of the hyaloid vascular system. Pictures were taken at 20× to show the red-boxed area. Scale bar in each picture indicates 100 μm. A↔P: anterior and posterior orientation of the eye.

To test whether the lens abnormalities in *Cited2* total knockout embryos are primary defects, Le-Cre was used to excise the *Cited2* gene in the mouse lens. Le-Cre is expressed in the surface ectoderm from embryonic day (E) 9.5 and in surface ectoderm derived structures including the lens, cornea, conjunctiva, and the eye lid. As expected, *Cited2* mRNA expression level was significantly decreased in six-week old *Cited2*^CKO^ mouse lens compared to control littermates, demonstrating the high deletion efficiency of Le-Cre. Since Cited2 is a negative regulator of HIF-1 and *VEGF* is one of HIF-1 target genes, we also examined the expression of *HIF-1α* and *VEGF* mRNAs. A modest increase of *HIF-1α* and a substantial increase of *VEGF* transcripts were observed in *Cited2*^CKO^ mouse lenses compared to those of control littermates (Figurer 1A). *Cited2*^CKO^ mice showed corneal opacity and the eyes were smaller than control littermates at six weeks of age. These mice also had lens stalks due to failed separation of the lens from the cornea and defective anterior chamber formation. In addition, abnormal retrolental tissue was observed in *Cited2*^CKO^ mouse eyes and lenses from these mice lost their transparency (Figure 1B). Immunostaining showed that Cited2 protein was mainly expressed in the nuclei of lens epithelial cells in control mouse lenses (Figure 1C). Compared with strong expression of Cited2 in control littermate lenses, *Cited2*^CKO^ mice showed a very weak signal in the disorganized lens epithelial cells. To address cell types involved in abnormal HVS, immunotstaining was performed with antibodies against α-SMA for pericytes, F4/80 for macrophages, and CD31 for endothelial cells. In *Cited2*^CKO^ mouse eyes, positive signals were detected for these proteins in abnormal HVS of the lens (Figure 1D). These results indicate that Cited2 deficiency is the primary defect during lens HVS regression.

### HIF-1α controls HVS formation and regression mediated by *Cited2*

**Figure 2:**
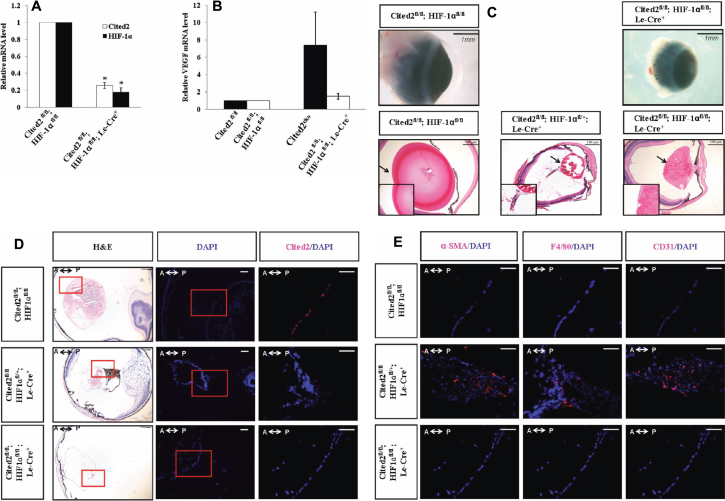
Le-Cre mediated deletion of *Cited2* and *HIF-1α* and phenotypic rescue by *HIF-1α* deletion. **A**: The expression levels of *Cited2* and *HIF-1α* were decreased in *Cited2*^fl/fl^;*HIF*^fl/fl^;Le-Cre^+^ mice compared to the control (n=3, *p<0.05). **B**: Increased *VEGF* in *Cited2*^CKO^ resumed to the normal level after *HIF-1α* was deleted in *Cited2*^CKO^ mice. **C**: Under dissecting microscope, eyes collected from *Cited2*^fl/fl^;*HIF*^fl/fl^;Le-Cre^+^ mice showed smaller sizes and cornea opacity (upper Panel). H&E staining showed smaller lens and lens stalk formation at the anterior part of the lens in *Cited2*^fl/fl^;*HIF*^fl/+^;Le-Cre^+^ and *Cited2*^fl/fl^;*HIF*^fl/fl^;Le-Cre^+^ mice. The insets represent higher magnification of the areas indicated by arrows. **D**: Expression level of Cited2 (magenta) was low in *Cited2*^fl/fl^;*HIF*^fl/fl^;Le-Cre^+^ mice compared to the control . H&E pictures were taken at 5× magnification to show the entire eye structure. DAPI pictures were taken at 10× using adjacent sections. Red box indicates the area shown in the counterstained pictures (20×). Scale bar in each picture indicates 100 μm. **E**: α-SMA, F4/80, and CD31 protein expression indicated the cell types in the hyaloid vascular system. Pictures were taken at 20× to show the red-boxed area. Scale bar in each picture indicates 100 μm. A↔P: anterior and posterior orientation of the eye.

To address the mechanism of *Cited2* deletion-induced abnormal hyaloid vasculature, our study focused on *HIF-1* and its target gene, *VEGF*. Deletion of *Cited2* and *HIF-1α* by Le-Cre resulted in lower expression levels of *HIF-1α* in *Cited2*^fl/fl^;*HIF-1α*^fl/+^ ;Le-Cre^+^ and *Cited2*^fl/fl^;*HIF-1α*^fl/fl^;Le-Cre^+^ mice compared to control mice (Figure 2A). The *VEGF* expression level was high in *Cited2*^fl/fl^;Le-Cre^+^ mouse lens but decreased to the normal level in *Cited2*^fl/fl^;*HIF-1α*^fl/fl^;Le-Cre^+^ mouse lens (Figure 2B). These results indicate that the deletion efficiency of *Cited2* and *HIF-1α* by Le-Cre was sufficient to study phenotypes rescued by deletion of *HIF-1α*. Eyes from *Cited2*^fl/fl^;*HIF-1α*^fl/fl^;Le-Cre^+^ showed corneal and lens opacity with a smaller size than control littermates at six weeks of age (Figure 2C). These mice showed lens stalk formation and abnormal retrolental tissues. Interestingly, abnormal retrolental tissues disappeared in *Cited2*^fl/fl^;*HIF-1α* ^fl/fl^;Le-Cre^+^ mouse eyes even though lens stalks persisted. This is consistent with our previous result that deletion of *HIF-1α* in *Cited2* knockout embryonic lens specifically eliminates abnormal retrolental tissues without affecting the corneal-lenticular stalk phenotype [12]. Immunostaining for Cited2 showed decreased Cited2 protein expression in *Cited2*^fl/fl^;*HIF-1α*^fl/fl^;Le-Cre^+^ mouse lens compared to controls (Figure 2D). Most *Cited2*^fl/fl^;*HIF-1α*^fl/+^;Le-Cre^+^ mice expressed α-SMA, CD31, and F4/80 in the posterior lens indicating the presence of the pericytes, endothelial cells, and macrophage cell types (Figure 2E).

**Figure 3:**
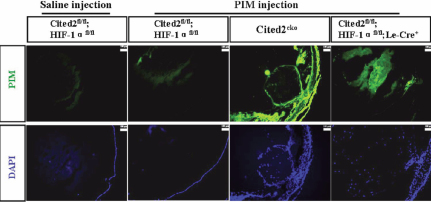
PIM staining for the hypoxic region. Eyes from different genotypes were stained with antibodies against PIM. PIM signal (Green) was strong in *Cited2*^CKO^ compared to the control. Eyes collected from *Cited2*^fl/fl^;*HIF*^fl/fl^;Le-Cre^+^ showed a weaker signal compared to *Cited2*^CKO^ mice. Saline injection was used as a negative control.

To detect the level of local hypoxia in the absence of Cited2 or both Cited2 and HIF-1α, we injected mice with pimonidazole (PIM) [27], a chemical that interacts with macromolecules under hypoxia, and sacrificed mice 2 h later. By immunostaining, we observed strong PIM signals in *Cited2*^CKO^ mouse lens compared to the control mouse lens and the lens from the negative control mouse injected with saline. Deletion of *HIF-1α* in *Cited2*^CKO^ mice (*Cited2*^fl/fl^;*HIF-1α*^fl/fl^;Le-Cre^+^) showed a weaker PIM signal compared to the *Cited2*^fl/fl^;Le-Cre^+^ mouse lens (Figure 3). This is consistent with our previous finding that, unlike wild type embryos, *Cited2*-deficient embryos remained highly hypoxic at 15.5 days post coitum (d.p.c.) in defective cardiac regions and both the severe hypoxia and cardiovascular defects were absent when the gene dosage of *HIF-1α* was reduced in *Cited2*-deficient embryos [11].

To compare the rescuing efficiency of *HIF-1α* deficiency, six-week old *Cited2*^CKO^ and *Cited2*^fl/fl^;*HIF-1α*^fl/+^;Le-Cre^+^ and *Cited2*^fl/fl^;*HIF-1α*^fl/fl^;Le-Cre^+^ mice were analyzed and number of mice in each group with abnormal HVS was determined (Table 1). All *Cited2*^CKO^ mice (100%) showed abnormal HVS formation in the lens. When one allele of *HIF-1α* was deleted in *Cited2*^CKO^ mice (*Cited2*^fl/fl^;*HIF-1α*^fl/+^;Le-Cre^+^), 71.4% of the mice showed abnormal HVS. Interestingly, when both alleles of *HIF-1α* were deleted in *Cited2*^CKO^ mice (*Cited2*^fl/fl^;*HIF-1α*^fl/fl^;Le-Cre^+^), the number of mice with abnormal HVS decreased to 27.3%.

**Table 1:** Number of mice with aberrant HVS formation.

**Genotypes**	**Age (weeks)**	**Lens stalk**	**Abnormal HVS**
Cited2^fl/fl^	6	0/8 (0%)	0/8 (0%)
Cited2^fl/fl^;HIF-1α^fl/+^/Cited2^fl/fl^;HIF-1α^fl/fl^	6	0/15 (0%)	0/15 (0%)
Cited2^cko^	6	7/7 (100%)	7/7 (100%)
Cited2^fl/fl^;HIF-1α^fl/+^; LeCre^+^	6	5/5 (100%)	5/7 (71.4%)
Cited2^fl/fl^;HIF-1α^fl/fl^; LeCre^+^	6	11/11 (100%)	3/11 (27.3%)
Cited2^fl/fl^;VEGF^fl/+^; LeCre^+^	6	4/4 (100%)	4/4 (100%)
Cited2^fl/fl^;VEGF^fl/fl^; LeCre^+^	6	10/10 (100%)	7/10 (70%)

### Deletion of *VEGF* partially rescues the HVS defects in *Cited2* deficient lens

**Figure 4:**
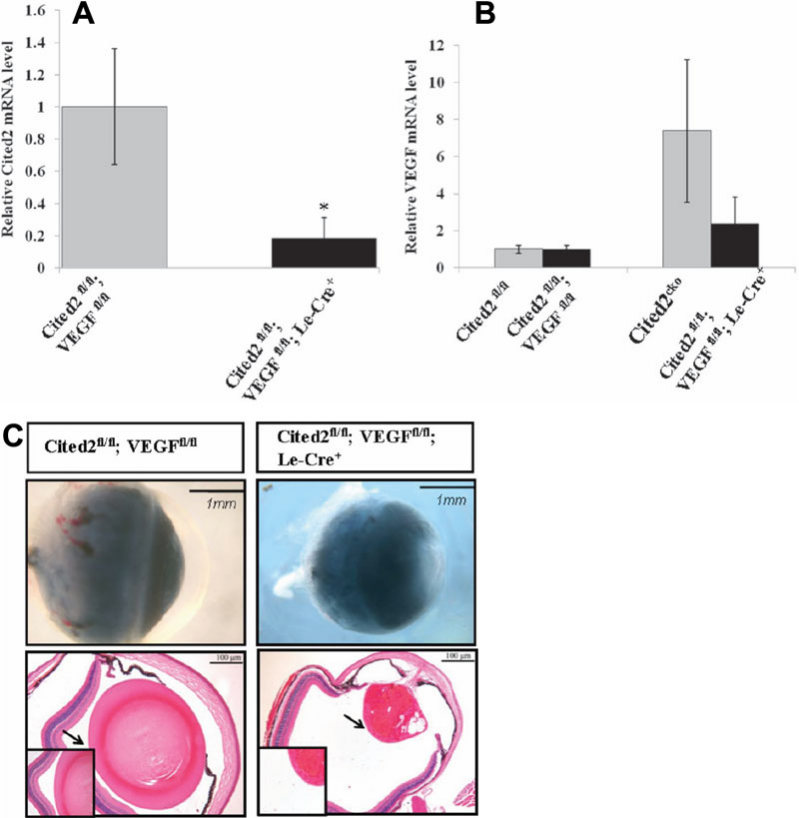
Le-Cre mediated deletion of *Cited2* and *VEGF* and phenotypic rescue by *VEGF* deletion. The expression level of *Cited2* was decreased in *Cited2*^fl/fl^;*VEGF*^fl/fl^;Le-Cre^+^ mouse lens compared to the control (n=3, *p<0.05). **B**: Relative *VEGF* mRNA expression was decreased in *Cited2*^fl/fl^;*VEGF*^fl/fl^;Le-Cre^+^ mouse lens compared to *Cited2*^CKO^. **C**: Morphological changes were similar between *Cited2*^CKO^ and *Cited2*^fl/fl^;*VEGF*^fl/fl^;Le-Cre^+^ by gross examination or under dissecting microscope (upper panel). The insets represent higher magnification of the areas indicated by arrows.

We previously observed increased mRNA expression of *VEGF*, a HIF-1 target gene, in *Cited2* deficient lens [12]. To answer whether VEGF plays a role in the formation of abnormal hyaloid vasculature induced by *Cited2* deficiency, we generated *Cited2*^fl/fl^;*VEGF*^fl/fl^;Le-Cre^+^ mice. Relative mRNA expression levels of *Cited2* (Figure 4A) and *VEGF* (Figure 4B) were decreased in *Cited2*^fl/fl^;*VEGF*^fl/fl^;Le-Cre^+^ mouse lens compared to controls. Most of *Cited2*^fl/fl^;*VEGF*^fl/+^;Le-Cre^+^ and *Cited2*^fl/fl^;*VEGF*^fl/fl^;Le-Cre^+^ mice showed a similar phenotype with the *Cited2*^CKO^ mice, including smaller eyes, lens stalk formation, lens opacity, and abnormal hyaloid formation (Figure 4C). Abnormal hyaloid vasculature was observed in 100% (4/4) *Cited2*^fl/fl^;*VEGF*^fl/+^;Le-Cre^+^ and 70% (7/10) *Cited2*^fl/fl^;*VEGF*^fl/fl^;Le-Cre^+^ mouse lenses (Figure 4C; Table 1), although the rescuing efficiency was not as high as with *HIF-1α* deletion. These results suggest that deletion of *VEGF* partially rescues the phenotype induced by *Cited2* deletion but that other HIF-1 downstream genes besides *VEGF* may be involved.

### *Cited2*^CKO^ mouse can be used as a mouse model for testing HIF-1α inhibitors

**Figure 5:**
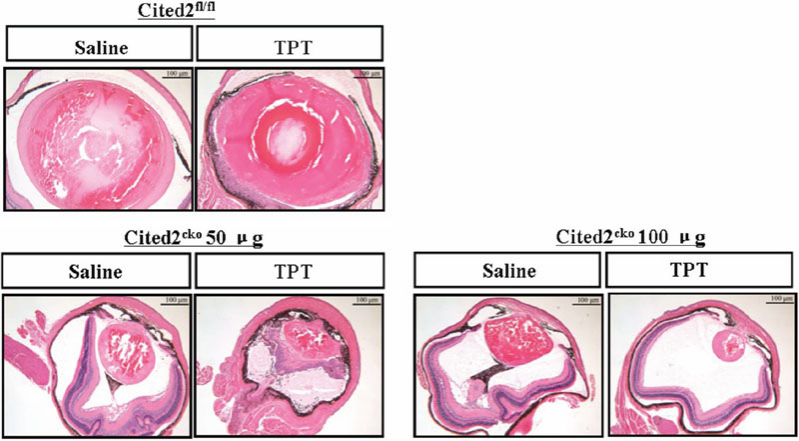
Intravitreal injection of a HIF-1α inhibitor in *Cited2*^CKO^ mice. Six-eight weeks old mice were received intravitreal injection of Topotecan (TPT). Fifty or 100 μg of TPT was injected into the left eye of *Cited2*^CKO^ mouse. Saline was injected into right eye as a negative control. Representative pictures are shown for 50 μg and 100 μg of TPT injection.

Since deletion of *HIF-1α* significantly rescued *Cited2* deficiency induced abnormal HVS in mouse lens, we tested whether the *Cited2*^CKO^ mouse could be used as a mouse model for testing potential HIF-1 inhibitors. Six- to eight-week old wild type or *Cited2*^CKO^ mice received once intravitreal injection 50 μg or 100 μg of Topotecan (TPT), a compound that inhibits the synthesis of HIF-1α, into the left eye [28]. Saline was injected into the right eye as a negative control. Three weeks after injection, eyes were collected and analyzed by standard histological methods. 20% (1/5) of mice with 50 μg of TPT injection showed less severe HVS and 25% (2/8) of mice with 100 μg of TPT injection showed improved HVS compared to saline injected right eyes (Figure 5). In addition, TPT injection did not affect lens stalk formation in the *Cited2*^CKO^ mouse. These results suggest that TPT injection partially eliminated abnormal HVS in the posterior lens in *Cited2*^CKO^ mouse.

## Discussion

*Cited2* deficient embryos exhibit several developmental defects and die in mid- to late gestation [9,12,29-35]. Although *Cited2* knockout embryos showed lens abnormalities [12], the mechanisms attributed to abnormal HVS was not fully addressed. Our results in this study clearly demonstrate that the lens abnormalities observed in *Cited2* null embryos are primary defects of *Cited2* deficiency in part through the deregulation of the HIF-1 pathway.

Previous studies have shown that intraocular injection of PIM at different stages of development detected adduct formation in all the stages examined, indicating that the lens exists in a chronically hypoxic state throughout embryonic development [27]. Our results showed that the lens from *Cited2*^CKO^ mouse exists in a highly hypoxic environment compared to the normal lens. After deletion of *HIF-1α* in *Cited2*^CKO^ mice, the hypoxic levels of the lens reached normal levels. It indicated that Le-Cre specific deletion of *Cited2* affects the oxygen tension in the lens, which is consistent with our finding in the *Cited2* knockout embryonic heart [11] and further support our hypothesis that Cited2 is a negative regulator of HIF-1.

The transcriptional regulator HIF-1 is the master mediator in the process of oxygen sensing and homeostasis. Under hypoxia, cell proliferation is inhibited by stabilized HIF-1 in cultured cells. Based on the fact that the lens is a hypoxic organ during chick development and HIF-1α is highly expressed in lens epithelial cells at mid-gestation in mouse embryos, we tested the hypothesis that *Cited2* deficiency induced abnormal HVS in the lens is mediated by HIF-1α. In *Cited2*^CKO^ mice, deletion of *HIF-1α* partially rescued abnormal HVS in *Cited2*^CKO^ mice. The results are consistent with our previous data that *HIF-1α* homozygous deletion in the lens eliminates the aberrant HVS formation in *Cited2* knockout embryonic eyes [12].

The potential role of VEGF in mediating the effect of a dysregulated Cited2-HIF-1 genetic interaction was suggested by previous studies in which we showed increased *VEGF* expression in *Cited2* deficient hearts and *HIF-1α* haploinsufficiency not only decreased *VEGF* expression but also rescued the heart defect in *Cited2* deficient embryos [11]. In the *Cited2* deficient embryonic lens, we also observed increased *VEGF* mRNA level. *VEGF* is one of the HIF-1 target genes that contributes to the formation of the vasculature in wounded tissues and tumors. Murine VEGF exists in three isoforms of 120, 164, and 188 amino acids [36]. Overexpression of specific *VEGF* isoforms results in different vascular patterning phenotypes: *VEGF120* transgenics show several vascular patterning defects in retinopathy of prematurity; *VEGF164–188* transgenics show features consistent with persistent hyperplastic primary vitreous (PHPV) [37,38]. Although *Cited2*^CKO^ mice showed higher expression levels of *VEGF* with aberrant formation of hyaloid vasculature in adult lens compared to normal mouse lens, it is not clear which isoform(s) are overexpressed and responsible for the vascular phenotype. Although the abnormalities were only partially corrected with deletion of *VEGF* in *Cited2* conditional knockout lens, it is consistent with a previous study by Shui et al. [16] that VEGF secreted by lens cells may stimulate the formation of the fetal vasculature, but regression of these vessels is not likely to be caused by a reduction in VEGF production by the lens. It is also possible that HIF-1 signaling could crosstalk with other regulators, such as TGFβ family cytokines. TGFβ2 has been shown to be an anti-angiogenic factor required for proper HVS remodeling during development since *TGFβ2* knockout mice display aberrant HVS formation, which is similar to the HVS phenotype in *Cited2* knockout eyes and can be rescued by overexpression of *TGFβ1*. It is possible that decreased expression of negative growth factors inhibiting angiogenesis, such as *TGFβ*, may also contribute to the HVS phenotypes observed in *Cited2*^CKO^ mice. Consistent with such a notion, we have previously shown that Cited2 interacts with Smads to modulate TGFβ signaling [39,40]. A more thorough expression profile analysis is necessary to identify possible cytokines and associated pathways other than VEGF that are involved.

Failed regression of HVS in *Cited2*^CKO^ lens also associates with smaller lens, lens opacity, and vitreous abnormality. These characteristics are similar to the pathological features of PHPV. Interestingly, PHPV has been diagnosed in a patient inflicted with von Hippel-Lindau disease, in which HIF-1 is overexpressed, suggesting a potential involvement of deregulated HIF-1 signaling in the pathogenesis of PHPV [41,42]. *Cited2* induced abnormal HVS is also similar to the phenotype in *Arf*^−/−^mice. *P19*^Arf^ may directly or indirectly alter pericyte biology through repression of *VEGF* to destabilize the underlying vessels [22]. Interestingly, several studies have suggested a possible link between *Arf* and *Cited2* in other cell types, although a formal connection in the ocular system has not been established [43,44].

In this study, the Cited2-HIF1α pathway in lens development was further confirmed by intravitreal injection of a HIF-1α inhibitor in *Cited2*^CKO^ mice. Topotecan (TPT), a drug that inhibits the synthesis of HIF-1α proteins, has been used in clinical trials in treating ovarian cancer and lung cancer. Periocular TPT in fibrin sealant can achieve volume reduction of small and recurrent retinoblastoma sufficient to allow successful focal therapy [45]. Our finding that intraocular injection of TPT eliminates aberrant vasculature in *Cited2*^CKO^ mice not only supports the hypothesis that the HIF-1 pathway is involved in abnormal HVS formation but also provides a novel mouse model for testing HIF-1 inhibitors. The major advantage of using this mouse line is that one does not have to generate tumor-bearing mice, which is time-consuming. In addition, only a small amount of inhibitor is needed for intravitreal injection, which is convenient for screening before a drug candidate is fully developed or synthesized. It is clear that TPT, like many HIF-1 inhibitors, is not a specific inhibitor. Since TPT did not affect lens stalk formation, it is likely that TPT did not induce general apoptosis in the injected lens, although more studies and the use of more specific HIF-1 inhibitors as they become available will be necessary to further validate our mouse model.

In conclusion, abnormal HVS formation in *Cited2*^CKO^ mice shows that the phenotype is a primary defect in the lens. In addition, deletion of *HIF-1α* or *VEGF* partially rescues the defect and HIF-1 inhibitor eliminates aberrant vasculature in *Cited2*^CKO^ mice. Based on these findings, *Cited2*^CKO^ mouse line could be used as a novel disease model for PHPV and for testing of potential HIF-1 inhibitors.
